# Histone deacetylases in Duchenne muscular dystrophy: a role in the mechanism of disease and a target for inhibition

**DOI:** 10.1186/s13148-025-02031-7

**Published:** 2025-12-04

**Authors:** Mariarita Bertoldi, Emilio Albamonte, Luca Bello, Adele D’amico, Riccardo Masson, Vincenzo Nigro, Marika Pane, Chiara Panicucci, Maria Sframeli, Federica Ricci

**Affiliations:** 1 Section of Biochemistry, Department of Neuroscience, Biomedicine and Movement Sciences, University of Verona, Verona, Italy; 2 The NEMO Clinical Center in Milan, Neurorehabilitation Unit, University of Milan, ASST Niguarda Hospital, 20162 Milan, Italy; 3 Department of Neurosciences DNS, University of Padova, Padua, Italy; 4 Unit of Neuromuscular and Neurodegenerative Disorders, Bambino Gesù Children’s Hospital IRCCS, 00163 Rome, Italy; 5 Department of Pediatric Neuroscience, Fondazione IRCCS Istituto Neurologico ‘Carlo Besta’, 20133 Milan, Italy; 6 Department of Precision Medicine, University of Campania “Luigi Vanvitelli”, Via Luigi de Crecchio 7, 80138 Naples, Italy; 7 Department of Life Science and Public Health, Pediatric Neurology, Catholic University of Sacred Heart, Rome, Italy; 8 Centre of Translational and Experimental Myology, IRCCS Istituto Giannina Gaslini, Genoa, Italy; 9 Department of Clinical and Experimental Medicine, University of Messina, 98122 Messina, Italy; 10 Neuromuscular Unit, Department of Neurosciences RLM, University of Torino, 10126 Turin, Italy

## Abstract

**Abstract:**

Aberrant activity of histone deacetylases (HDACs) is a pathological phenomenon in several diseases, including Duchenne muscular dystrophy (DMD). In DMD, the upregulation of HDACs is driven by the disassembly of the dystrophin-associated protein complex (DAPC), which, under normal physiological conditions, provides mechanical stability to muscle fibres and acts as a signalling hub anchoring signalling proteins and molecules to their functional sites. In dystrophic muscle, DAPC disassembly causes delocalisation of signalling proteins and, therefore, disrupts signalling pathways. Displacement of epigenetic signalling molecules leads to the uncontrolled activity of HDACs and excessive removal of acetyl groups from histone proteins. Consequently, chromatin becomes tightly bound, preventing the expression of genes involved in muscle homeostasis. The pathological consequences of increased HDAC activity extend beyond muscle fibres, affecting several cell types, translating into a chronically activated immune system, promoting fibrotic and adipose tissue formation and impairing muscle regeneration. Here, we review the current evidence implicating HDACs as a key driver in DMD disease development and progression. We describe the mechanism of HDAC overactivity and the downstream consequences that contribute to the pathogenesis of the disease by disrupting muscle repair and regeneration. Finally, we highlight HDACs as targets for inhibition, offering a novel therapeutic strategy to counteract the multiple pathological events in DMD.

**Graphical abstract:**

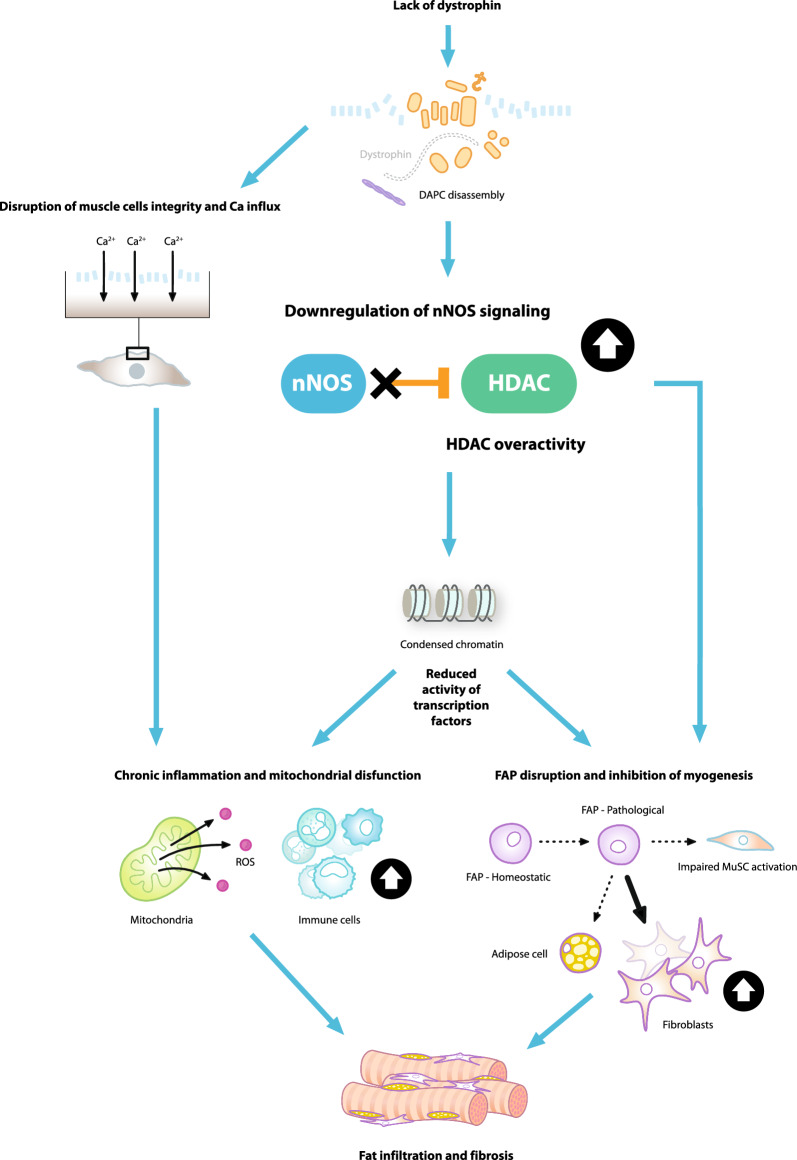

## Introduction

Duchenne muscular dystrophy (DMD) is a severe disease characterised by progressive loss of muscle tissues [1]. The earliest symptoms present after the first years of life, with loss of ambulation typically occurring in the early teens [2] and the need for assisted ventilation at approximately 20 years of age [3]. Premature death occurs in most patients from 20 to 40 years of age from cardiac and/or respiratory failure [4].

DMD is an X-linked recessive disorder caused by mutations in the dystrophin gene that lead to a complete or near-complete loss of functional dystrophin protein expression [1]. This loss of functional dystrophin leads to disassembly of the transmembrane dystrophin-associated protein complex (DAPC) [5, 6]. The disassembly of this complex triggers a cascade of pathological events that include chronic inflammation and failed regeneration [5, 6]. One key consequence of dystrophin loss and DAPC disassembly is increased histone deacetylase (HDAC) enzyme activity [7, 8].

HDACs are evolutionarily conserved enzymes involved in the epigenetic regulation of gene expression that remove acetylated groups from the lysine of histone proteins [9]. HDACs have been implicated in skeletal muscle remodelling, both in physiological and pathological conditions [10]. HDAC hyperactivity has been observed in DMD animal models and patients with DMD [7, 11–13]. In particular, DMD muscles display an aberrant, constitutive activation of HDAC2 [7]. As such, the inhibition of HDAC activity has received attention as a therapeutic approach for treating muscular dystrophies [14].

## DAPC disassembly leads to HDAC upregulation

### Multi-functional role of DAPC

The dystrophin protein is an essential component of the DAPC, a highly organised multiprotein complex that plays a pivotal role in muscle fibre structural integrity [15]. The DAPC comprises several proteins, including dystrophin, syntrophins, dystroglycans, sarcoglycans and neuronal nitric oxide synthase (nNOS) [15]. Dystrophin acts as a bridge to connect the intracellular cytoskeletal filaments to the sarcolemma, stabilising the sarcolemma during muscle fibre contraction and relaxation. However, the DAPC also plays a key role in cell signalling, acting as a molecular scaffold to anchor a variety of signalling molecules [16].

### DAPC signalling regulates HDAC activity

The muscle-specific nNOS enzyme, which produces intramuscular nitric oxide (NO) is anchored to the sarcolemma by the syntrophin subcomplex of the DAPC [17–19]. NO is a critical signalling molecule involved in regulating a variety of cellular processes involved in muscle tissue maintenance and repair [20]. For example, NO activates transient receptor potential cation channel, subfamily V, member 1 (TRPV1), increasing intracellular calcium and subsequently activating mammalian target of rapamycin (mTOR)-mediated signalling to drive muscle fibre growth [21].

Another key role of NO is to mediate the S-nitrosylation of HDACs via a post-translational modification where NO attaches a nitrosyl group to a cysteine residue in HDACs [7]. This modification inhibits the function of HDACs and plays a crucial role in regulating gene expression in muscle cells [7]. This gene regulation is dependent on the balance between the activities of histone acetyltransferases (HATs) and HDACs to essentially “switch on and off” target genes. HATs catalyse the addition of acetyl groups to histone proteins, inducing chromatin unravelling and triggering the transcription of genes coding for muscle repair factors. In contrast, HDACs remove acetyl groups from histones, rendering chromatin more compact and thereby inaccessible for transcription [20]. For example, HDAC2 inhibits follistatin gene transcription in mdx (dystrophin-deficient) muscle cells, which in turn blocks myostatin, an inhibitor of muscle growth [7, 22, 23]. It has also been suggested that HDAC2 effects on muscle could occur via epigenetic modulation of microRNA genes [24]. Decreased production of NO, therefore, leads to increased activity of HDACs, reductions of histone acetylation, more condensed chromatin structure and ultimately repressed expression of selected genes [20].

### DAPC disassembly triggers aberrant HDAC activity

In DMD, DAPC disassembly disrupts the physical connection between the sarcolemma, cytoskeleton and the extracellular matrix. This causes the sarcolemma to become leaky and highly susceptible to injury [6, 25]. DAPC disassembly also leads to the mislocalisation of nNOS and the subsequent depletion of NO [17, 18]. The reduction in NO generation prevents NO-mediated S-nitrosylation and inhibition of HDACs, leading to an aberrant, constitutive HDAC hyperactivation [7].

Patients with DMD exhibit selective alterations of nNOS and catalytic activity from muscle membranes, contributing to muscle fibre degeneration [17]. In dystrophic (mdx) mice, these alterations have been shown to impair contractile function and cause muscle fatigue [26, 27]. Additional effects of the disassembly of DAPC include elevated reactive oxygen species, mitochondrial dysfunction and calcium dysregulation [25, 28].

## The pathological consequences of HDAC hyperactivity

HDAC upregulation and consequent hyperactivity have been observed in a wide range of cell types and have a wide range of pathological consequences for muscle damage repair [10].

### Mitochondria and muscle fibres

Mitochondrial dysfunction is one of the earliest deficits in mouse models of DMD [29, 30]. It is associated with a progressive impairment of mitochondrial biogenesis caused by epigenetic modifications and increased histone deacetylation of the peroxisome proliferator-activated receptor-gamma coactivator 1 α (PGC-1α) promoter [31]. The progressive increase in HDAC activity in DMD may explain the progressive deacetylation of the PGC-1a promoter [31]. Administration of the HDAC inhibitors givinostat and trichostatin A was shown to restore the physiological epigenetic profile on the PGC-1α promoter, improving mitochondrial biogenesis and enabling a positive fibre-type switch towards a more oxidative phenotype [31].

Fast-twitch fibres are preferentially affected in DMD, while slow-twitch (oxidative) fibres are relatively spared [32], suggesting that shifting skeletal muscle towards a slower, more oxidative phenotype may protect dystrophic muscles. Muscle fibre-type shifts are regulated by the HDAC-myocyte enhancer factor 2 (MEF2)-PGC-1α pathway [33, 34]. Class II HDACs act as repressors of MEF2, a key transcriptional activator for PGC-1α expression in skeletal muscle [33, 34]. Thus, HDAC inhibition may promote a transition to a slow/oxidative fibre type [33, 34].

### Immune cells

Immune cell infiltration is a main feature of DMD and is strongly associated with disease severity [25]. In healthy muscle cells, pro-inflammatory macrophages clear damaged fibres after injury. These cell types are then replaced by anti-inflammatory macrophages that drive fibre regeneration [35]. The transition from a pro- to anti-inflammatory state is driven by regulatory T (Treg) cells (Foxp3 + Treg) [36]. In DMD, repeated injury causes the chronic inflammatory response to be maintained by overlapping pro-inflammatory and anti-inflammatory signalling, preventing the full resolution of inflammation [25].

HDACs are known to play a significant role in modulating immune responses, through epigenetic effects on various transcription factors. These include transcription factor nuclear factor kappa B (NF-κB), a master regulator of innate immune responses and essential for many macrophage functions [37–39], and Foxp3, the master transcription factor of Treg cells [39, 40]. Thus, the HDAC hyperactivity observed in DMD might be expected to affect the balance between pro-inflammatory and anti-inflammatory immune cell populations [41]. Of note, in addition to its role in modulating immune responses, the transcription factor NF-κB also plays a key role in various aspects of skeletal muscle biology [42], representing another means by which HDAC dysregulation may contribute to the pathophysiology of DMD.

### Fibro-adipogenic cells/muscle satellite cells

Muscle satellite cells (MuSCs) are the principal contributors to the regeneration of injured muscles. MuSCs are skeletal muscle precursor cells that are activated in response to stress stimuli, such as injury [43, 44]. Activated MuSCs undergo asymmetric division and myogenic differentiation to repair muscle [43, 44]. HDACs regulate gene expression in MuSCs by controlling the activity of myogenic proteins such as MyoD and MEF2 [43, 45, 46], and so the hyperactivation of HDACs in DMD can impair the differentiation of satellite cells [43, 47].

Fibro-adipogenic cells (FAPs) also play an important role in muscle regeneration [48]. FAPs enhance the activation and differentiation of MuSCs to repair damaged muscle [49]. However, in DMD, FAPs differentiate primarily into fibroblasts or fat cells [50]. Studies in mdx mice have shown that HDACs determine the fate of FAPs via regulation of myogenic microRNAs [51]. In young mdx mice, HDAC inhibition was shown to induce the expression of core components of the myogenic transcriptional machinery, resulting in promyogenic differentiation while suppressing the fibro-adipogenic phenotype [51].

Therefore, both the altered differentiation process in the MuSCs and the loss of myogenic signals from FAPs contribute to making the MuSCs ineffective during the muscle repair and regeneration process in patients with DMD.

## HDAC inhibition is a novel therapeutic approach in DMD

Although HDAC inhibitors have been in development for many years across several diseases, relatively few have been approved for use [14]. The discovery that the lack of dystrophin and DAPC disassembly results in constitutive HDAC activation in DMD muscles has led to efforts to explore HDAC inhibitors as a treatment strategy for DMD [14]. Considering that muscle damage and repair mechanisms are disrupted by overactive HDAC activity, suppressing this activity could enhance muscle repair and reduce the severity of DMD pathology.

Early studies in rodent and human muscle cells indicated that HDAC inhibitors could enhance muscle differentiation through follistatin expression and the recruitment and fusion of myoblasts into preformed myotubes [52, 53]. A subsequent seminal study showed that HDAC inhibitors increased the size of myofibres in dystrophin-deficient (mdx) mice by inducing the expression of the myostatin antagonist follistatin in satellite cells [23]. These studies inspired the clinical translation of HDAC inhibitors for the treatment of DMD.

Givinostat is a class I and II HDAC inhibitor that counteracts the pathogenic events downstream of dystrophin deficiency [54]. It has been approved in the United States, United Kingdom and Europe for the treatment of DMD in patients aged 6 years and older, regardless of the underlying genetic mutation [55–57]. Its potential therapeutic effects were first established in various preclinical studies. Givinostat was shown to reduce the production of pro-inflammatory cytokines in human peripheral blood mononuclear cells, and to exhibit anti-inflammatory effects in a mouse model of inflammation [58]. In mouse models of DMD, givinostat promoted the formation of muscles with increased cross-sectional area and reduced fibrosis and fatty infiltration, leading to improvements of muscle function as evaluated by treadmill and grip strength tests [59, 60].

Givinostat has shown a beneficial effect in patients with DMD over the course of several clinical trials. In a Phase 1 trial in healthy males, givinostat reduced the production of pro-inflammatory cytokines without affecting production of anti-inflammatory cytokines, with an accompanying dose-dependent, transient reduction in platelets [61]. In an open-label Phase 2 study investigating the effects of givinostat on histological disease progression in boys with DMD, treatment with givinostat significantly increased muscle fibre area fraction while reducing fibrosis, necrosis and fat deposition [62]. The EPIDYS Phase 3 study met its primary endpoint, with a significantly smaller decline in the time taken to perform the four-stair climb with givinostat (plus corticosteroids) compared with control (placebo plus corticosteroids), and showed favourable efficacy across secondary endpoints [63]. As measured by magnetic resonance spectroscopy, givinostat reduced new muscle fat fraction infiltration in the vastus lateralis by approximately 30% [63] and also significantly reduced new fat infiltration in all key muscle groups required for ambulation compared with control [64].

Studies of other HDAC inhibitors are ongoing. A recent study demonstrated beneficial effects of the HDAC6-selective inhibitor tubastatin A in the mdx mouse [65]. Inhibition of HDAC6 by tubastatin A was shown to increase muscle strength, improve microtubule and neuromuscular junction organisation and reduce muscle atrophy in mdx mice [65]. Rather than directly affecting the expression of individual genes as shown for other HDACs, HDAC6 acts on transforming growth factor-β signalling by targeting Smad3 in the cytoplasm [65]. Epigenetic small molecule screening in *dmd* zebrafish has also identified HDAC inhibitor candidates for further evaluation in DMD [66, 67].

## Conclusions

DMD is an irreversible, progressive muscle-wasting disease caused by mutations in the dystrophin gene. Dystrophin is a core component of the DAPC, connecting actin filaments within muscle cells to the sarcolemma and acting as a shock absorber that stabilises the membrane during muscle contraction. Dystrophin regulates muscle repair, at least in part, via HDAC activity control. HDAC upregulation or hyperactivity is a major contributor to the pathology of DMD and, therefore, represents a therapeutic avenue in the treatment of DMD and possibly other neuromuscular diseases. HDAC inhibition influences pathophysiological and epigenetic mechanisms contributing to DMD and can have an important role structurally in recovering muscle fibre integrity, and biochemically in restoring signalling pathways.

For these reasons, the inhibition of HDAC offers a novel approach to slow down the progression of the disease with the aim to: (i) re-establish the production of anti-inflammatory proteins to prevent the immune system from being constantly activated and, thus, reduce chronic inflammation; (ii) reduce fibrosis and fat deposition by redirecting FAPs differentiation towards a myogenic path rather than differentiating into fibroblasts and adipose cells; (iii) promote muscle regeneration by restoring the ability of FAPs to produce growth factors needed to activate muscle stem cells which transform into new mature muscle fibres or repair damaged fibres. An improved understanding of the pathophysiological role of HDAC in DMD will provide insights into how pharmacological treatments can counteract the multiple and complex pathological events in DMD.

## Data Availability

Not applicable. No datasets were generated or analyzed during the current study.

## References

[1] Birnkrant DJ, Bushby K, Bann CM, Apkon SD, Blackwell A, Brumbaugh D, et al. Diagnosis and management of Duchenne muscular dystrophy, part 1: diagnosis, and neuromuscular, rehabilitation, endocrine, and gastrointestinal and nutritional management. Lancet Neurol. 2018;17(3):251–67. 29395989 10.1016/S1474-4422(18)30024-3 PMC5869704

[2] Bello L, Morgenroth LP, Gordish-Dressman H, Hoffman EP, McDonald CM, Cirak S. DMD genotypes and loss of ambulation in the CINRG Duchenne natural history study. Neurology. 2016;87(4):401–9. 27343068 10.1212/WNL.0000000000002891 PMC4977110

[3] Birnkrant DJ, Bushby K, Bann CM, Alman BA, Apkon SD, Blackwell A, et al. Diagnosis and management of Duchenne muscular dystrophy, part 2: respiratory, cardiac, bone health, and orthopaedic management. Lancet Neurol. 2018;17(4):347–61. 29395990 10.1016/S1474-4422(18)30025-5 PMC5889091

[4] Landfeldt E, Thompson R, Sejersen T, McMillan HJ, Kirschner J, Lochmüller H. Life expectancy at birth in Duchenne muscular dystrophy: a systematic review and meta-analysis. Eur J Epidemiol. 2020;35(7):643–53. 32107739 10.1007/s10654-020-00613-8 PMC7387367

[5] Dowling P, Swandulla D, Ohlendieck K. Cellular pathogenesis of Duchenne muscular dystrophy: progressive myofibre degeneration, chronic inflammation, reactive myofibrosis and satellite cell dysfunction. Eur J Transl Myol. 2023;33(4):11856. 37846661 10.4081/ejtm.2023.11856 PMC10811648

[6] Kodippili K, Rudnicki MA. Satellite cell contribution to disease pathology in Duchenne muscular dystrophy. Front Physiol. 2023;14:1180980. 37324396 10.3389/fphys.2023.1180980 PMC10266354

[7] Colussi C, Mozzetta C, Gurtner A, Illi B, Rosati J, Straino S, et al. HDAC2 blockade by nitric oxide and histone deacetylase inhibitors reveals a common target in Duchenne muscular dystrophy treatment. Proc Natl Acad Sci USA. 2008;105(49):19183–7. 19047631 10.1073/pnas.0805514105 PMC2614736

[8] Consalvi S, Tucciarone L, Macrì E, De Bardi M, Picozza M, Salvatori I, et al. Determinants of epigenetic resistance to HDAC inhibitors in dystrophic fibro-adipogenic progenitors. EMBO Rep. 2022;23(6):e54721. 35383427 10.15252/embr.202254721 PMC9171680

[9] Milazzo G, Mercatelli D, Di Muzio G, Triboli L, De Rosa P, Perini G, et al. Histone deacetylases (HDACs): evolution, specificity, role in transcriptional complexes, and pharmacological actionability. Genes. 2020;11(5):556. 32429325 10.3390/genes11050556 PMC7288346

[10] Sandonà M, Cavioli G, Renzini A, Cedola A, Gigli G, Coletti D, et al. Histone deacetylases: molecular mechanisms and therapeutic implications for muscular dystrophies. Int J Mol Sci. 2023. 10.3390/ijms24054306. 36901738 10.3390/ijms24054306 PMC10002075

[11] Renzini A, Marroncelli N, Cavioli G, Di Francescantonio S, Forcina L, Lambridis A, et al. Cytoplasmic HDAC4 regulates the membrane repair mechanism in Duchenne muscular dystrophy. J Cachexia Sarcopenia Muscle. 2022;13(2):1339–59. 35170869 10.1002/jcsm.12891 PMC8977968

[12] Spreafico M, Cafora M, Bragato C, Capitanio D, Marasca F, Bodega B, et al. Targeting HDAC8 to ameliorate skeletal muscle differentiation in Duchenne muscular dystrophy. Pharmacol Res. 2021;170:105750. 34214631 10.1016/j.phrs.2021.105750

[13] Colussi C, Gurtner A, Rosati J, Illi B, Ragone G, Piaggio G, et al. Nitric oxide deficiency determines global chromatin changes in Duchenne muscular dystrophy. FASEB J. 2009;23(7):2131–41. 19264835 10.1096/fj.08-115618

[14] Mozzetta C, Sartorelli V, Steinkuhler C, Puri PL. HDAC inhibitors as pharmacological treatment for Duchenne muscular dystrophy: a discovery journey from bench to patients. Trends Mol Med. 2024;30(3):278–94. 38408879 10.1016/j.molmed.2024.01.007 PMC11095976

[15] Canessa EH, Spathis R, Novak JS, Beedle A, Nagaraju K, Bello L, et al. Characterization of the dystrophin-associated protein complex by mass spectrometry. Mass Spectrom Rev. 2024;43(1):90–105. 36420714 10.1002/mas.21823

[16] Constantin B. Dystrophin complex functions as a scaffold for signalling proteins. Biochim Biophys Acta. 2014;1838(2):635–42. 24021238 10.1016/j.bbamem.2013.08.023

[17] Brenman JE, Chao DS, Xia H, Aldape K, Bredt DS. Nitric oxide synthase complexed with dystrophin and absent from skeletal muscle sarcolemma in Duchenne muscular dystrophy. Cell. 1995;82(5):743–52. 7545544 10.1016/0092-8674(95)90471-9

[18] Chang WJ, Iannaccone ST, Lau KS, Masters BS, McCabe TJ, McMillan K, et al. Neuronal nitric oxide synthase and dystrophin-deficient muscular dystrophy. Proc Natl Acad Sci USA. 1996;93(17):9142–7. 8799168 10.1073/pnas.93.17.9142 PMC38609

[19] Lai Y, Thomas GD, Yue Y, Yang HT, Li D, Long C, et al. Dystrophins carrying spectrin-like repeats 16 and 17 anchor nNOS to the sarcolemma and enhance exercise performance in a mouse model of muscular dystrophy. J Clin Invest. 2009;119(3):624–35. 19229108 10.1172/JCI36612 PMC2648692

[20] Tidball JG, Wehling-Henricks M. Nitric oxide synthase deficiency and the pathophysiology of muscular dystrophy. J Physiol. 2014;592(21):4627–38. 25194047 10.1113/jphysiol.2014.274878 PMC4253467

[21] Ito N, Ruegg UT, Kudo A, Miyagoe-Suzuki Y, Takeda S. Activation of calcium signaling through Trpv1 by nNOS and peroxynitrite as a key trigger of skeletal muscle hypertrophy. Nat Med. 2013;19(1):101–6. 23202294 10.1038/nm.3019

[22] Abe S, Soejima M, Iwanuma O, Saka H, Matsunaga S, Sakiyama K, et al. Expression of myostatin and follistatin in mdx mice, an animal model for muscular dystrophy. Zoolog Sci. 2009;26(5):315–20. 19715499 10.2108/zsj.26.315

[23] Minetti GC, Colussi C, Adami R, Serra C, Mozzetta C, Parente V, et al. Functional and morphological recovery of dystrophic muscles in mice treated with deacetylase inhibitors. Nat Med. 2006;12(10):1147–50. 16980968 10.1038/nm1479

[24] Cacchiarelli D, Martone J, Girardi E, Cesana M, Incitti T, Morlando M, et al. MicroRNAs involved in molecular circuitries relevant for the Duchenne muscular dystrophy pathogenesis are controlled by the dystrophin/nNOS pathway. Cell Metab. 2010;12(4):341–51. 20727829 10.1016/j.cmet.2010.07.008

[25] Bez Batti Angulski A, Hosny N, Cohen H, Martin AA, Hahn D, Bauer J, et al. Duchenne muscular dystrophy: disease mechanism and therapeutic strategies. Front Physiol. 2023;14:1183101. 37435300 10.3389/fphys.2023.1183101 PMC10330733

[26] Rebolledo DL, Kim MJ, Whitehead NP, Adams ME, Froehner SC. Sarcolemmal targeting of nNOSμ improves contractile function of mdx muscle. Hum Mol Genet. 2016;25(1):158–66. 26604149 10.1093/hmg/ddv466 PMC4690500

[27] Froehner SC, Reed SM, Anderson KN, Huang PL, Percival JM. Loss of nNOS inhibits compensatory muscle hypertrophy and exacerbates inflammation and eccentric contraction-induced damage in mdx mice. Hum Mol Genet. 2015;24(2):492–505. 25214536 10.1093/hmg/ddu469 PMC4275075

[28] Allen DG, Whitehead NP, Froehner SC. Absence of dystrophin disrupts skeletal muscle signaling: roles of Ca2+, reactive oxygen species, and nitric oxide in the development of muscular dystrophy. Physiol Rev. 2016;96(1):253–305. 26676145 10.1152/physrev.00007.2015 PMC4698395

[29] Vila MC, Rayavarapu S, Hogarth MW, Van der Meulen JH, Horn A, Defour A, et al. Mitochondria mediate cell membrane repair and contribute to Duchenne muscular dystrophy. Cell Death Differ. 2017;24(2):330–42. 27834955 10.1038/cdd.2016.127 PMC5299714

[30] Hughes MC, Ramos SV, Turnbull PC, Rebalka IA, Cao A, Monaco CMF, et al. Early myopathy in Duchenne muscular dystrophy is associated with elevated mitochondrial H(2) O(2) emission during impaired oxidative phosphorylation. J Cachexia Sarcopenia Muscle. 2019;10(3):643–61. 30938481 10.1002/jcsm.12405 PMC6596403

[31] Giovarelli M, Zecchini S, Catarinella G, Moscheni C, Sartori P, Barbieri C, et al. Givinostat as metabolic enhancer reverting mitochondrial biogenesis deficit in Duchenne muscular dystrophy. Pharmacol Res. 2021;170:105751. 34197911 10.1016/j.phrs.2021.105751

[32] Webster C, Silberstein L, Hays AP, Blau HM. Fast muscle fibers are preferentially affected in Duchenne muscular dystrophy. Cell. 1988;52(4):503–13. 3342447 10.1016/0092-8674(88)90463-1

[33] Cohen TJ, Choi MC, Kapur M, Lira VA, Yan Z, Yao TP. HDAC4 regulates muscle fiber type-specific gene expression programs. Mol Cells. 2015;38(4):343–8. 25728750 10.14348/molcells.2015.2278 PMC4400309

[34] Potthoff MJ, Wu H, Arnold MA, Shelton JM, Backs J, McAnally J, et al. Histone deacetylase degradation and MEF2 activation promote the formation of slow-twitch myofibers. J Clin Invest. 2007;117(9):2459–67. 17786239 10.1172/JCI31960 PMC1957540

[35] Tidball JG, Villalta SA. Regulatory interactions between muscle and the immune system during muscle regeneration. Am J Physiol Regul Integr Comp Physiol. 2010;298(5):R1173–87. 20219869 10.1152/ajpregu.00735.2009 PMC2867520

[36] Burzyn D, Kuswanto W, Kolodin D, Shadrach JL, Cerletti M, Jang Y, et al. A special population of regulatory T cells potentiates muscle repair. Cell. 2013;155(6):1282–95. 24315098 10.1016/j.cell.2013.10.054 PMC3894749

[37] Ashburner BP, Westerheide SD, Baldwin AS Jr. The p65 (RelA) subunit of NF-kappaB interacts with the histone deacetylase (HDAC) corepressors HDAC1 and HDAC2 to negatively regulate gene expression. Mol Cell Biol. 2001;21(20):7065–77. 11564889 10.1128/MCB.21.20.7065-7077.2001 PMC99882

[38] Zhong H, May MJ, Jimi E, Ghosh S. The phosphorylation status of nuclear NF-kappa B determines its association with CBP/p300 or HDAC-1. Mol Cell. 2002;9(3):625–36. 11931769 10.1016/s1097-2765(02)00477-x

[39] Licciardi PV, Karagiannis TC. Regulation of immune responses by histone deacetylase inhibitors. ISRN Hematol. 2012;2012:690901. 22461998 10.5402/2012/690901 PMC3313568

[40] Beier UH, Akimova T, Liu Y, Wang L, Hancock WW. Histone/protein deacetylases control Foxp3 expression and the heat shock response of T-regulatory cells. Curr Opin Immunol. 2011;23(5):670–8. 21798734 10.1016/j.coi.2011.07.002 PMC3190028

[41] Aartsma-Rus A. Histone deacetylase inhibition with givinostat: a multi-targeted mode of action with the potential to halt the pathological cascade of Duchenne muscular dystrophy. Front Cell Dev Biol. 2025;12:1514898. 39834392 10.3389/fcell.2024.1514898 PMC11743666

[42] Peterson JM, Bakkar N, Guttridge DC. NF-κB signaling in skeletal muscle health and disease. Curr Top Dev Biol. 2011;96:85–119. 21621068 10.1016/B978-0-12-385940-2.00004-8

[43] Rugowska A, Starosta A, Konieczny P. Epigenetic modifications in muscle regeneration and progression of Duchenne muscular dystrophy. Clin Epigenet. 2021;13(1):13. 10.1186/s13148-021-01001-z PMC7814631 33468200

[44] Mukund K, Subramaniam S. Skeletal muscle: a review of molecular structure and function, in health and disease. Wiley Interdiscip Rev Syst Biol Med. 2020;12(1):e1462. 31407867 10.1002/wsbm.1462 PMC6916202

[45] Puri PL, Iezzi S, Stiegler P, Chen TT, Schiltz RL, Muscat GE, et al. Class I histone deacetylases sequentially interact with MyoD and pRb during skeletal myogenesis. Mol Cell. 2001;8(4):885–97. 11684023 10.1016/s1097-2765(01)00373-2

[46] McKinsey TA, Zhang CL, Olson EN. Activation of the myocyte enhancer factor-2 transcription factor by calcium/calmodulin-dependent protein kinase-stimulated binding of 14-3-3 to histone deacetylase 5. Proc Natl Acad Sci USA. 2000;97(26):14400–5. 11114197 10.1073/pnas.260501497 PMC18930

[47] Marrone AK, Shcherbata HR. Dystrophin orchestrates the epigenetic profile of muscle cells via miRNAs. Front Genet. 2011;2:64. 22303359 10.3389/fgene.2011.00064 PMC3268617

[48] Mozzetta C, Consalvi S, Saccone V, Tierney M, Diamantini A, Mitchell KJ, et al. Fibroadipogenic progenitors mediate the ability of HDAC inhibitors to promote regeneration in dystrophic muscles of young, but not old mdx mice. EMBO Mol Med. 2013;5(4):626–39. 23505062 10.1002/emmm.201202096 PMC3628105

[49] Joe AW, Yi L, Natarajan A, Le Grand F, So L, Wang J, et al. Muscle injury activates resident fibro/adipogenic progenitors that facilitate myogenesis. Nat Cell Biol. 2010;12(2):153–63. 20081841 10.1038/ncb2015 PMC4580288

[50] Uezumi A, Ito T, Morikawa D, Shimizu N, Yoneda T, Segawa M, et al. Fibrosis and adipogenesis originate from a common mesenchymal progenitor in skeletal muscle. J Cell Sci. 2011;124(Pt 21):3654–64. 22045730 10.1242/jcs.086629

[51] Saccone V, Consalvi S, Giordani L, Mozzetta C, Barozzi I, Sandoná M, et al. HDAC-regulated myomiRs control BAF60 variant exchange and direct the functional phenotype of fibro-adipogenic progenitors in dystrophic muscles. Genes Dev. 2014;28(8):841–57. 24682306 10.1101/gad.234468.113 PMC4003277

[52] Iezzi S, Cossu G, Nervi C, Sartorelli V, Puri PL. Stage-specific modulation of skeletal myogenesis by inhibitors of nuclear deacetylases. Proc Natl Acad Sci USA. 2002;99(11):7757–62. 12032356 10.1073/pnas.112218599 PMC124343

[53] Iezzi S, Di Padova M, Serra C, Caretti G, Simone C, Maklan E, et al. Deacetylase inhibitors increase muscle cell size by promoting myoblast recruitment and fusion through induction of follistatin. Dev Cell. 2004;6(5):673–84. 15130492 10.1016/s1534-5807(04)00107-8

[54] McDonald CM, Guglieri M, Vučinić D, Acsadi G, Brandsema JF, Bruno C, et al. Long-term evaluation of givinostat in Duchenne muscular dystrophy, and natural history comparisons. Ann Clin Transl Neurol. 2025. 10.1002/acn3.70165. 40830818 10.1002/acn3.70165 PMC12623843

[55] US Food and Drug Administration. DUVYZAT (givinostat) oral suspension, Label. Available at https://www.accessdata.fda.gov/2024.

[56] European Medicines Agency. Duvyzat oral suspension, Summary of Product Characteristics. Available at https://www.ema.europa.eu/en/medicines/human/EPAR/duvyzat2025.

[57] UK Medicines and Healthcare products Regulatory Agency. Duvyzat oral suspension, Summary of Product Characteristics. Available at https://products.mhra.gov.uk/2024.

[58] Leoni F, Fossati G, Lewis EC, Lee JK, Porro G, Pagani P, et al. The histone deacetylase inhibitor ITF2357 reduces production of pro-inflammatory cytokines in vitro and systemic inflammation in vivo. Mol Med. 2005;11(1–12):1–15. 16557334 10.2119/2006-00005.Dinarello PMC1449516

[59] Consalvi S, Mozzetta C, Bettica P, Germani M, Fiorentini F, Del Bene F, et al. Preclinical studies in the mdx mouse model of Duchenne muscular dystrophy with the histone deacetylase inhibitor givinostat. Mol Med. 2013;19(1):79–87. 23552722 10.2119/molmed.2013.00011 PMC3667212

[60] Licandro SA, Crippa L, Pomarico R, Perego R, Fossati G, Leoni F, et al. The pan HDAC inhibitor givinostat improves muscle function and histological parameters in two Duchenne muscular dystrophy murine models expressing different haplotypes of the LTBP4 gene. Skelet Muscle. 2021;11(1):19. 34294164 10.1186/s13395-021-00273-6 PMC8296708

[61] Furlan A, Monzani V, Reznikov LL, Leoni F, Fossati G, Modena D, et al. Pharmacokinetics, safety and inducible cytokine responses during a phase 1 trial of the oral histone deacetylase inhibitor ITF2357 (givinostat). Mol Med. 2011;17(5–6):353–62. 21365126 10.2119/molmed.2011.00020 PMC3105139

[62] Bettica P, Petrini S, D’Oria V, D’Amico A, Catteruccia M, Pane M, et al. Histological effects of givinostat in boys with Duchenne muscular dystrophy. Neuromuscul Disord. 2016;26(10):643–9. 27566866 10.1016/j.nmd.2016.07.002

[63] Mercuri E, Vilchez JJ, Boespflug-Tanguy O, Zaidman CM, Mah JK, Goemans N, et al. Safety and efficacy of givinostat in boys with Duchenne muscular dystrophy (EPIDYS): a multicentre, randomised, double-blind, placebo-controlled, phase 3 trial. Lancet Neurol. 2024;23(4):393–403. 38508835 10.1016/S1474-4422(24)00036-X

[64] Vandenborne K. Givinostat in DMD: results of the EPIDYS study with particular attention to MR measures of muscle fat fraction. Muscular Dystrophy Association Clinical & Scientific Conference; 19–22 March, 2023; Dallas, TX, USA, 2023.

[65] Osseni A, Ravel-Chapuis A, Belotti E, Scionti I, Gangloff YG, Moncollin V, et al. Pharmacological inhibition of HDAC6 improves muscle phenotypes in dystrophin-deficient mice by downregulating TGF-β via Smad3 acetylation. Nat Commun. 2022;13(1):7108. 36402791 10.1038/s41467-022-34831-3 PMC9675748

[66] Louie KW, Hasegawa EH, Farr GH 3rd, Ignacz AC, Paguio A, Maenza A, et al. Epigenetic small molecule screening identifies a new HDACi compound for ameliorating Duchenne muscular dystrophy. Mol Ther Nucleic Acids. 2025;36(3):102683. 40917897 10.1016/j.omtn.2025.102683 PMC12410445

[67] Farr GH 3rd, Morris M, Gomez A, Pham T, Kilroy E, Parker EU, et al. A novel chemical-combination screen in zebrafish identifies epigenetic small molecule candidates for the treatment of Duchenne muscular dystrophy. Skelet Muscle. 2020;10(1):29. 33059738 10.1186/s13395-020-00251-4 PMC7559456

